# Preparation and investigation of hydrolyzed polyacrylonitrile as a preliminary biomedical hydrogel

**DOI:** 10.1186/s40824-015-0043-1

**Published:** 2015-10-26

**Authors:** Ji Hoon Park, Guo Zhe Tai, Bo Keun Lee, Seung Hun Park, Ja Yong Jang, Jung Soo Lee, Jae Ho Kim, Kwideok Park, Ju Woong Jang, Moon Suk Kim

**Affiliations:** Department of Molecular Science and Technology, Ajou University, Suwon, 443-759 South Korea; R&DB Center, Cellumed Co., Ltd., Seoul, 153-803 South Korea; Center for Biomaterials, Korea Institute of Science and Technology, Seoul, 136-791 South Korea

**Keywords:** Hydrolyzed polyacrylonitrile, Radical polymerization, Swelling, Hydrogel, Biomedical applications

## Abstract

**Background:**

Hydrolyzed polyacrylonitrile (HPAN) ha**s** attracted much attention as a hydrogel for a broad range of biomedical applications**.** Therefore, in this study, we prepared HPAN derivatives with controllable compositions by the radical polymerization of acrylonitrile (AN), methacrylic acid (MAA) and *N*-isopropylacrylamide (NIPAM) monomers.

**Results:**

The prepared poly(AN-co-MAA-co-NIPAM) copolymers had different ratios of AN, MAA, and NIPAM and molecular weights ranging from 2000 to 50,000. The copolymers were prepared as films to examine their properties. The prepared copolymer films showed different solubilities, contact angles, and swelling ratios. The properties of the copolymer films were affected by the hydrophobic PAN segments and the hydrophilic PMAA or PNIPAM segments.

**Conclusion:**

Thus, we conclude that introducing PMAA and PNIPAM segments with different ratios and lengths into PAN segments could represent a method of controlling the hydrogel properties of copolymers.

## Background

Hydrogels have been developed extensively for a broad range of biomedical applications [1, 2]. Most types of hydrogels can absorb large quantities of water relative to their initial weight because of their intrinsic hydrophilicity. Because of this propensity to retain large amounts of water, hydrogels can be used in biomedical applications in dehydrated and/or hydrated form. Hydrogels have been applied extensively as smart polymers for drug carriers, contact lens materials, and orthopedic implants [3–7]. In vivo, hydrogels expand to form swelled shapes by absorbing body-derived fluids [8–11].

Many hydrogels have been developed using various polymers. Among them, partial hydrolyzed polyacrylonitrile (HPAN) is produced through a chemical reaction of polyacrylonitrile (PAN) with sodium hydroxide [12–14]. The reaction produces a water-soluble HPAN block copolymer consisting of hydrophobic nitrile functional groups and hydrophilic poly(acrylic acid), partially neutralized poly(acrylic acid), and poly(acrylamide) [15–18].

HPAN mainly expands in water and/or in body-derived fluids to form swollen hydrogels that are highly biocompatible and biodurable and cause minimal inflammation following implantation. Uniquely, HPAN shows elasticity and tensile strength that are very similar to those of body tissues, such as cartilage and the nucleus pulposus of the intervertebral disc [19–21]. Thus, HPAN has been developed extensively for minimally invasive spine surgery [22].

Although HPAN has a significant advantage in biomedical applications, control over the hydrophilic and hydrophobic segments is limited by the hetero-chemical reaction of PAN with sodium hydroxide and/or amine. Thus, developing a simple preparation method for HPAN copolymers with controllable composition has been the subject of practical development efforts.

HPAN derivatives can be easily prepared using acrylonitrile (AN), methacrylic acid (MAA) and *N*-isopropylacrylamide (NIPAM) monomers, which are cheap and easy to polymerize. The prepared poly(AN–co-MAA-co-NIPAM) copolymer derivatives have the ability to absorb higher water and form hydrogel, which is an essential criterion for a hydrogel suitable for biomedical applications.

To the best of our knowledge, few previous studies have addressed the preparation of HPAN derivatives with controllable compositions. Thus, in this work, we prepared HPAN derivatives with controllable compositions of AN, MAA and NIPAM by radical polymerization. The solution and swelling properties of the copolymer were also examined for hydrogel application.

## Methods

### Materials

Acrylonitrile (AN), methacrylic acid (MAA), and *N*-isopropylacrylamide (NIPAM) were purchased from Aldrich (MO, USA) and distilled over CaH_2_ under reduced pressure. Azobisisobutyronitrile (AIBN) was recrystallized in methanol. THF was purchased from Burdick & Jackson (MI, USA). The HPLC-grade *n*-hexane and ethyl ether were purchased from Samchun (Pyeongtaek, Korea) and used as received.

### Synthesis of poly(AN-co-MAA-co-NIPAM)

All glassware was dried by heating in vacuum and handled under a dry nitrogen stream. The typical polymerization process to produce poly(AN-co-MAA-co-NIPAM) with a AN/MAA/NIPAM ratio of 50/25/25 and a molecular weight of 2000 is as follows: AIBN (0.43 g, 2.6 mmol) and THF (40 mL) were introduced into a flask. Then, AN (1.39 g, 26.2 mmol), MAA (1.13 g, 13.1 mmol), and NIPAM (1.48 g, 13.1 mmol) were added by syringe and bubbled with dry nitrogen for 30 min to remove oxygen from the reaction solution. The solution was stirred vigorously at 75 °C under a dry nitrogen atmosphere. After stirring for 16 h, the reaction mixture was poured into ethyl ether to precipitate the copolymer, which was separated from the supernatant by decantation and then filtered. The resulting polymer was dried under vacuum to yield the copolymers. The copolymers are summarized in according to their feed compositions Table 1. ^1^H-NMR and ^13^C-NMR spectra were measured using a Varian Mercury Plus 400 system with DMSO-*d*_6_ in the presence of tetramethylsilane. The AN/MAA/NIPAM ratios in the poly(AN-co-MAA-co-NIPAM) copolymers were determined from the ^13^C-NMR spectra by comparing the average integration value of the characteristic carbonyl signal of AN/MAA/NIPAM.

**Table 1:** Preparation and swelling ratios of poly(AN-co-MAA-co-NIPAM), poly(AN-co-MAA) and poly(AN-co-NIPAM) copolymers

	Molar ratio (%) (AN-MAA-NIPAM)	Molar ratio (%) (AN-MAA-NIPAM)^a^	M_w_	Yield (%)	Swelling ratio (%)	Solubility (mg/mL)
P(AN-MAA-NIPAM)	50:25:25	47:23:30	2 000	89	65	12
	25:50:25	25:52:23	2 000	61	72	14
	25:25:50	22:24:54	2 000	90	81	21
	50:25:25	49:26:25	20 000	82	62	5
	50:25:25	47:24:29	50 000	62	58	~1
P(AN-MAA)	50:50:0	53:47:0	2 000	93	60	10
P(AN-NIPAM)	50:0:50	46:0:54	2 000	94	68	17

^a^Determined by ^13^C-NMR

### Synthesis of poly(AN-co-MAA) and poly(AN-co-NIPAM)

Poly(AN-co-MAA) with an AN/MAA ratio of 50/50 and poly(AN-co-NIPAM) with an AN/NIPAM ratio of 50/50 were prepared using same copolymerization method as described in the previous section.

### Determination of solution properties

250 mg of the poly(AN-co-MAA-co-NIPAM), poly(AN-co-MAA) and poly(AN-co-NIPAM) copolymers were introduced into 5-mL vials. Two milliliter of distilled water (pH 7), a solution of pH 3, and a solution of pH 10 (Adjusted to the desire pH with 1 N HCl and NaOH) were added to the vials and incubated for 1 h and observed. The solubility of poly(AN-co-MAA-co-NIPAM), poly(AN-co-MAA) and poly(AN-co-NIPAM) copolymers in DW was determined at 37 °C.

### Preparation of copolymer films

Poly(AN-co-MAA-co-NIPAM), poly(AN-co-MAA) and poly(AN-co-NIPAM) films were prepared using solvent casting. One milligram of copolymers was solubilized in 1 mL of THF. The solution was casted on polyethylene film and allowed to dry slowly at 10 °C for 2 d. Next, the casted films were dried in a vacuum oven at room temperature for 4 days, resulting in smooth and non-porous films. The copolymer films were cut into discs with 6 mm diameters and 200 μm thicknesses.

### Contact angle measurement of copolymer films

The water contact angle was measured using the sessile drop method at room temperature with an optical bench-type contact angle goniometer (Phoenix 150, SEO, Suwon, Korea). One drop of purified water (5 μL) was deposited onto the prepared film surface by means of a microsyringe. The water contact angle was measured within 5 s.

### Determination of swelling ratios

The completely dried poly(AN-co-MAA-co-NIPAM), poly(AN-co-MAA) and poly(AN-co-NIPAM) films were placed in 5-mL vials, and 1 mL of PBS at 37 °C was added. The swollen films were removed after 24 h, and the surface was quickly blotted free of water with filter paper. The films were then weighed and placed in the same bath. The mass measurements were continued until equilibrium was reached. The equilibrium swelling ratio was determined according to the conventional gravimetric method using the following equation: Swelling ratio (%) = [equilibrium swollen weight − initial dried weight) × 100] / [initial dried weight].

## Results and discussion

### Preparation of poly(AN-co-MAA-co-NIPAM), poly(AN-co-MAA) and poly(AN-co-NIPAM)

**Fig. 1:**
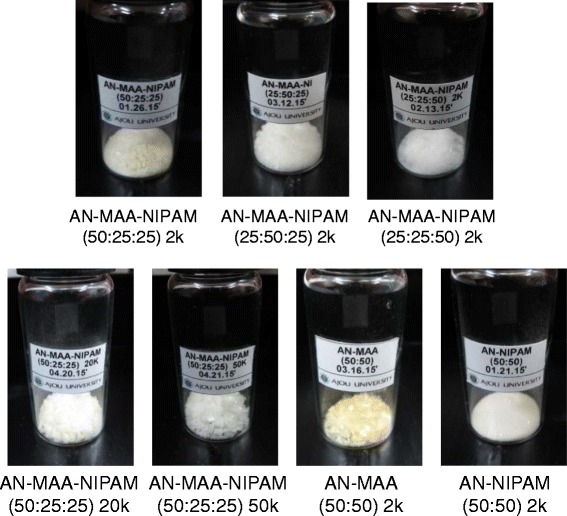
Scheme of polymers. Scheme of the polymerization of poly(AN-co-MAA-co-NIPAM) and pictures of the obtained poly(AN-co-MAA-co-NIPAM), poly(AN-co-MAA) and poly(AN-co-NIPAM) copolymers

The preparation of poly(AN-co-MAA-co-NIPAM), poly(AN-co-MAA) and poly(AN-co-NIPAM) copolymers is summarized in Table 1. All copolymers were synthesized through the radical polymerization of the monomers AN, MAA, and NIPAM using the AIBM as an initiator. All copolymers were obtained as colorless or slight yellowish copolymers after isolation by precipitation (Fig. 1). The poly(AN-co-MAA-co-NIPAM) copolymers were prepared with molecular weights ranging from 2000 to 50,000 using different ratios of AN, MAA, and NIPAM.

**Fig. 2:**
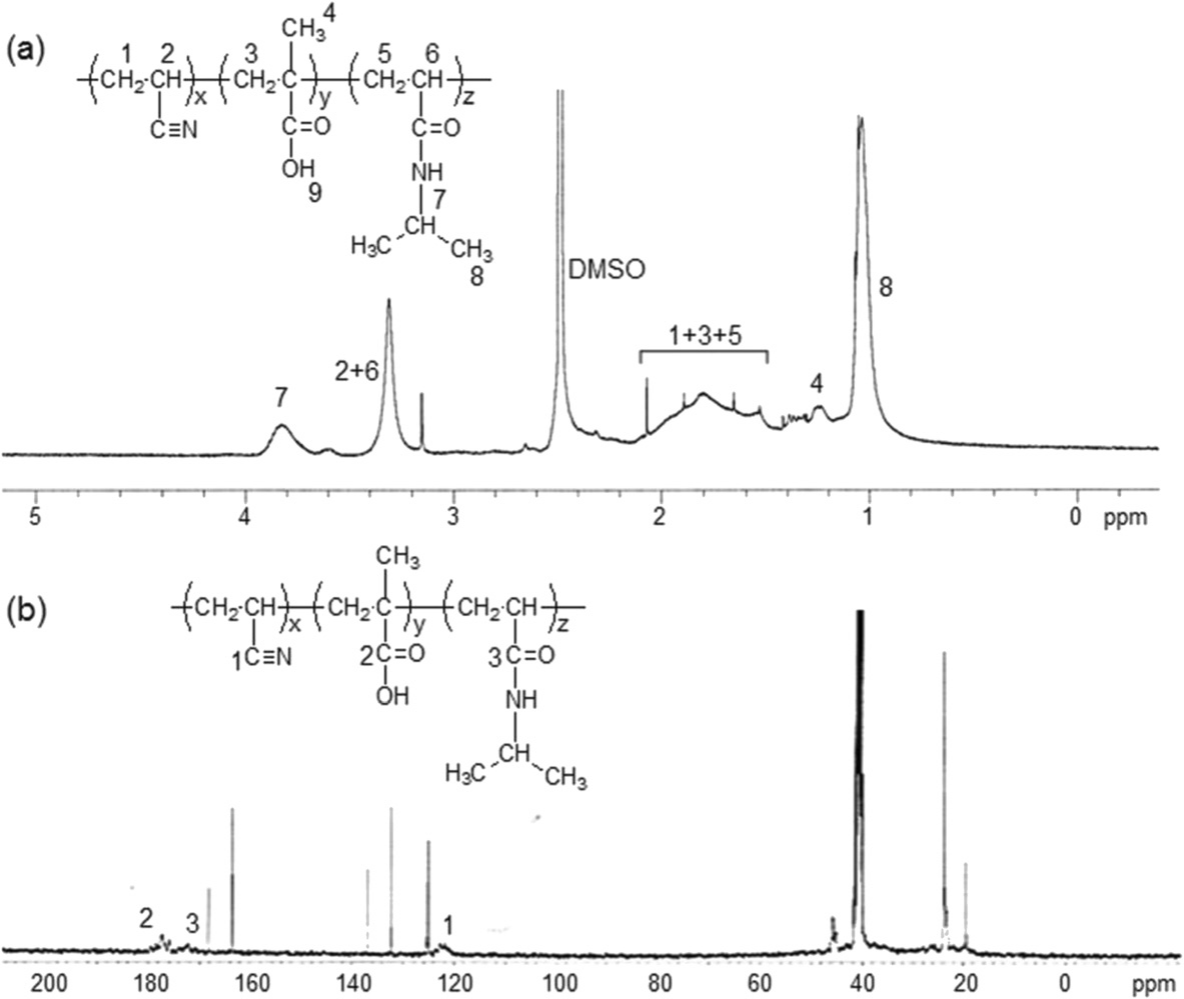
NMR spectra of polymer. **a** ^1^H-NMR and **b** ^13^C-NMR spectra of poly(AN-co-MAA-co-NIPAM) with a ratio of 50/25/25 in DMSO-*d*_6_

Figure 2 shows the ^1^H-NMR and ^13^C-NMR spectra of poly(AN-co-MAA-co-NIPAM). Poly(AN-co-MAA-co-NIPAM) copolymer exhibited characteristic peaks of PAN, PMAA, and PNIPAM. The methyl, methylene and methine protons were observed in ^1^H-NMR spectra. The carbons of the carbonyl groups in PAN, PMAA, and PNIPAM were observed at *δ* =122, 177, and 172 ppm in ^13^C-NMR spectra. The ratio of PAN, PMAA, and PNIPAM was determined according to the carbon integration ratios of the carbonyl groups, which agreed well with the feed ratio values. The poly(AN-co-MAA) and poly(AN-co-NIPAM) also showed characteristic peaks of PAN, PMAA, or PNIPAM, and the ratio of the prepared copolymers determined by ^13^C-NMR was also in good agreement with the feed ratio values as summarized in Table 1.

These findings indicated that HPAN derivatives with controllable compositions of AN-MAA-NIPAM were successfully prepared by radical polymerization. Thus, in this work, we have demonstrated that it is possible to prepare copolymers with distinct compositions, lengths and molecular weights of the hydrophobic PAN segment and the hydrophilic PMAA and/or PNIPAM segments.

### Solution properties of poly(AN-co-MAA-co-NIPAM), poly(AN-co-MAA) and poly(AN-co-NIPAM)

**Fig. 3:**
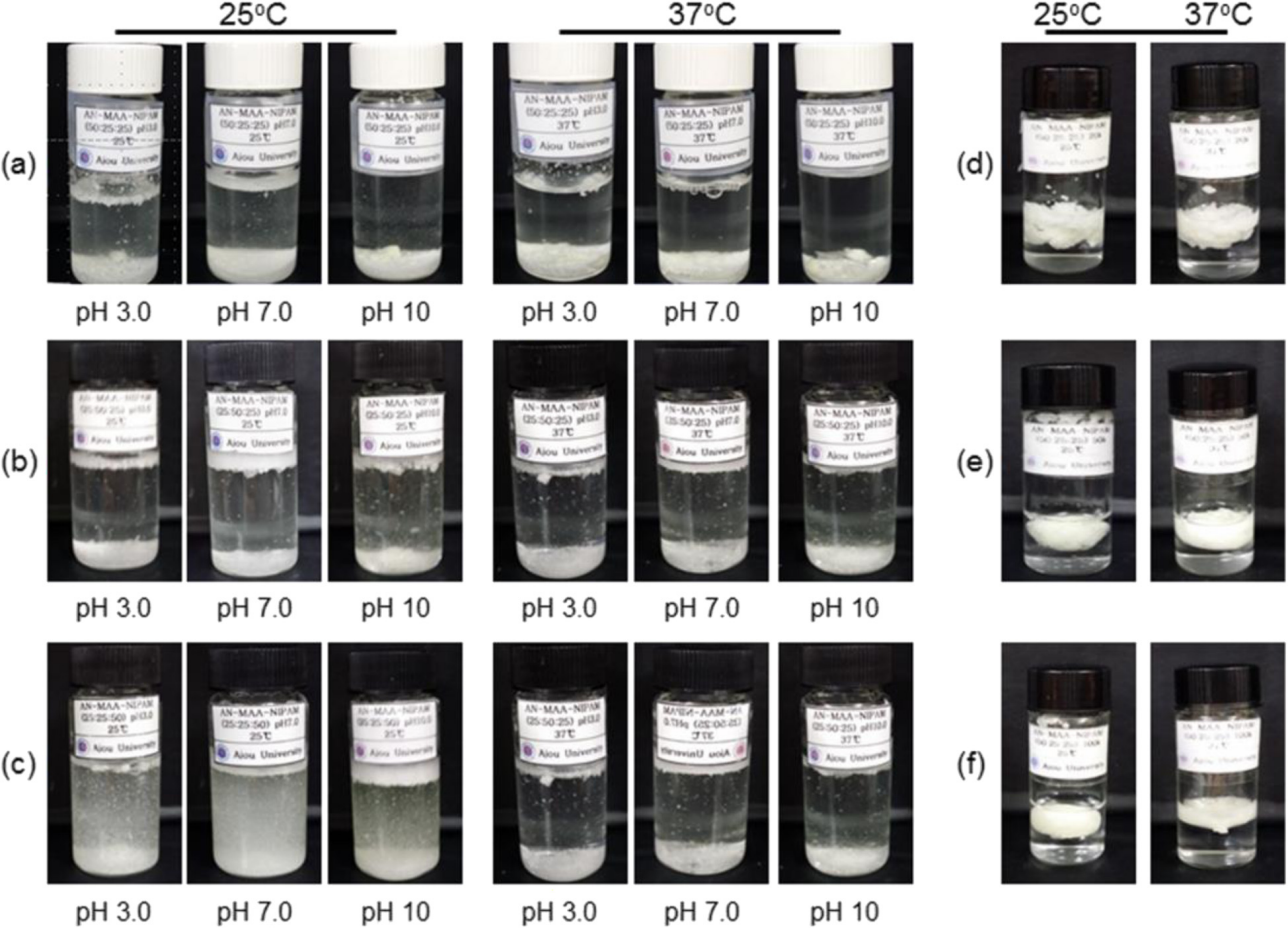
Pictures of polymer. Pictures of poly(AN-co-MAA-co-NIPAM), poly(AN-co-MAA) and poly(AN-co-NIPAM) copolymer solutions with ratios of (**a**) 50/25/25, (**b**) 25:50:25, and (**c**) 25:25:50 (AN:MAA:NIPAM) at pH 3.0, 7.0, and 10.0 and with molecular weights of (**d**) 2000, (**e**) 20,000 and **f** 50,000

To examine the solution properties of poly(AN-co-MAA-co-NIPAM), poly(AN-co-MAA) and poly(AN-co-NIPAM), the aqueous solutions of the copolymers were prepared by dissolving them in solutions of pH 3, 7, and 10. Figure 3 shows pictures of 10-wt% copolymers at pH 3, 7, and 10 at 25 °C and 37 °C. The copolymer solutions showed different solubility as summarized in Table 1. The solubility of poly(AN-co-MAA-co-NIPAM) with a ratio of 50/25/25 exhibited less solubility than those with ratios of 25/50/25 and 20/25/50 in similar molecular weights, which indicated that the solubility decreased as the amount of hydrophobic PAN segment increased. In addition, poly(AN-co-MAA-co-NIPAM) appeared to become nearly insoluble as the molecular weight increased from 2000 to 20,000 and 50,000. Furthermore, poly(AN-co-MAA-co-NIPAM) with a ratio of 20/25/50 exhibited solubility of 10 ~ 17 mg/mL. These findings indicate that the hydrophilic PNIPAM segment mainly affects the solution properties.

### Surface properties of poly(AN-co-MAA-co-NIPAM), poly(AN-co-MAA) and poly(AN-co-NIPAM)

**Fig. 4:**
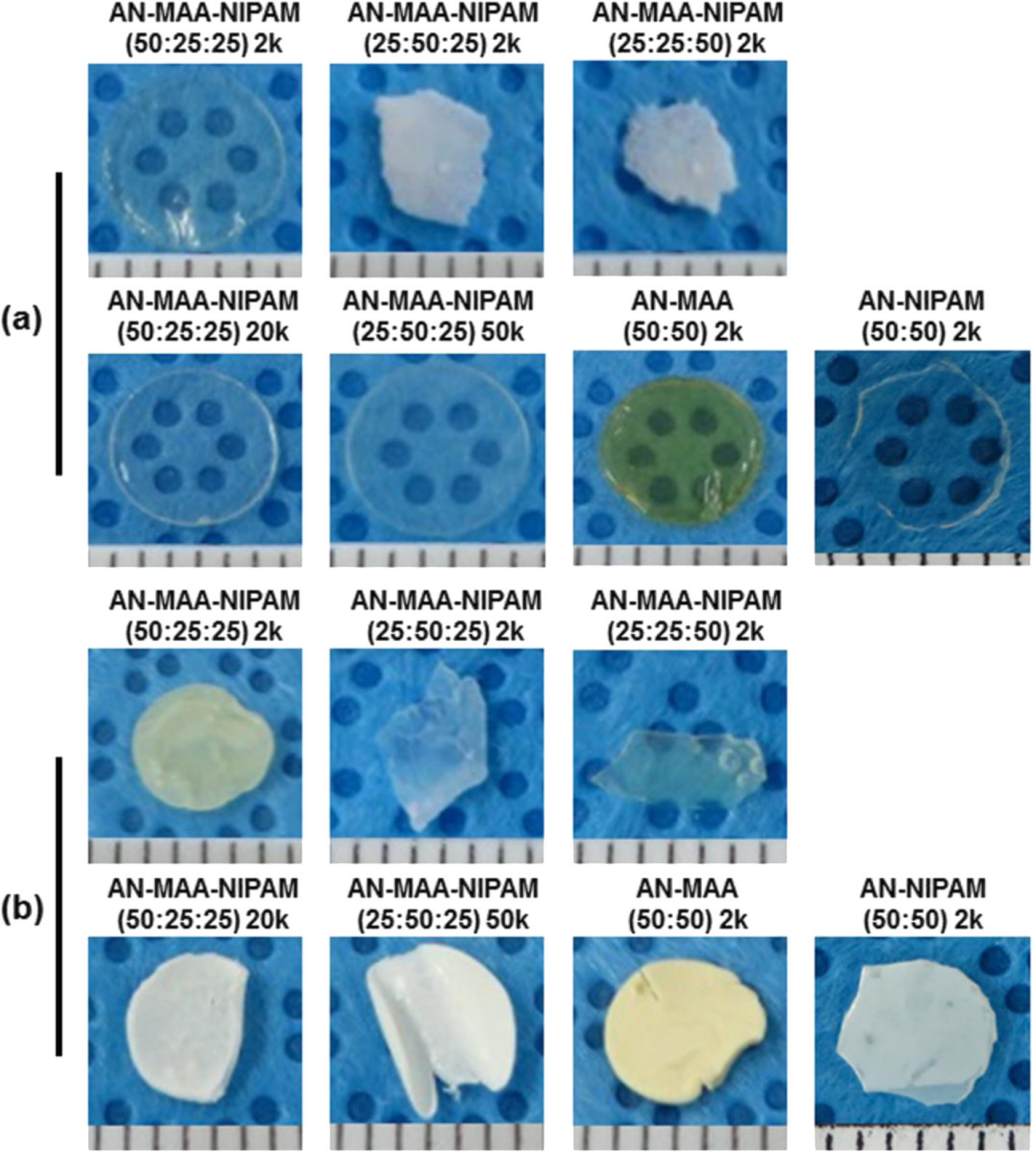
Images of polymer films. Images (**a**) before and (**b**) after swelling of the films prepared from poly(AN-co-MAA-co-NIPAM), poly(AN-co-MAA) and poly(AN-co-NIPAM)

Copolymer films were prepared to examine the surface properties of poly(AN-co-MAA-co-NIPAM), poly(AN-co-MAA) and poly(AN-co-NIPAM) (Fig. 4a). The poly(AN-co-MAA-co-NIPAM) and poly(AN-co-MAA) films with larger ratios of PAN exhibited a slight yellowish color. The films with larger ratios of NIPAM exhibited brittle properties.

**Fig. 5:**
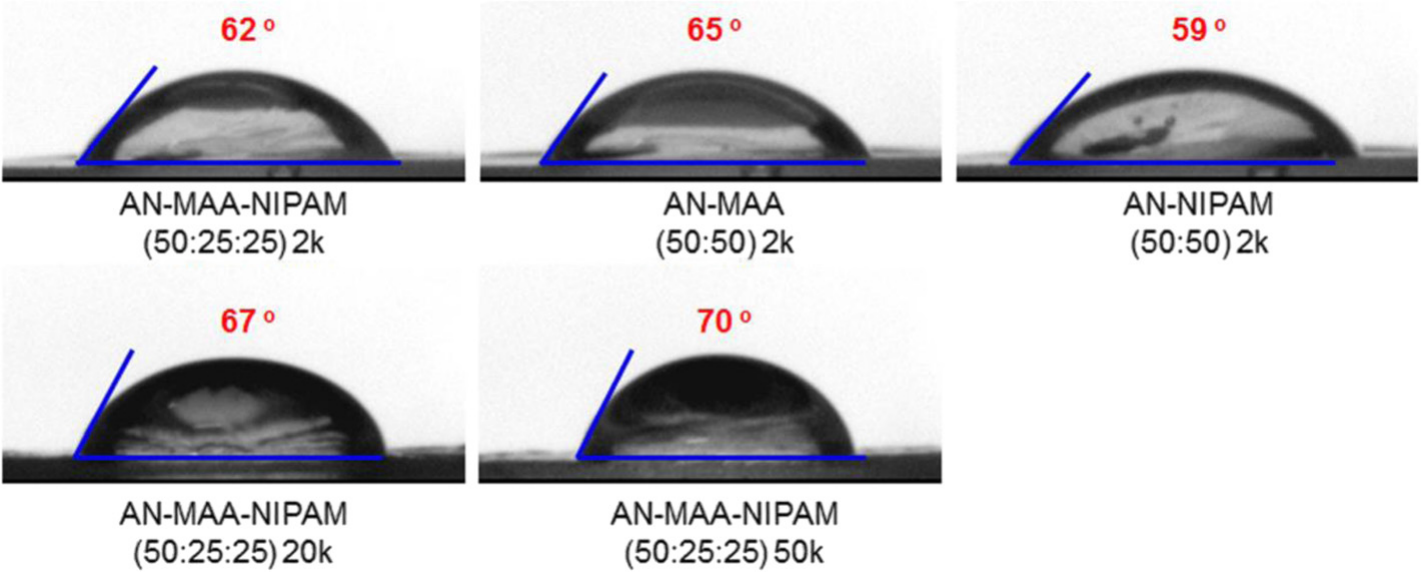
Contact angles of polymer films. Contact angles of the films prepared from poly(AN-co-MAA-co-NIPAM), poly(AN-co-MAA) and poly(AN-co-NIPAM)

As shown in Fig. 5, the contact angles of poly(AN-co-MAA-co-NIPAM) of 2000, 20,000 and 50,000 were 62, 67, and 70° respectively, indicating slight increasing of contact angles as molecular weight increased. Meanwhile poly(AN-co-MAA) exhibited slight increasing to angles of 65° and poly(AN-co-NIPAM) slightly decreased to 59°.

### Swelling properties of poly(AN-co-MAA-co-NIPAM), poly(AN-co-MAA) and poly(AN-co-NIPAM)

**Fig. 6:**
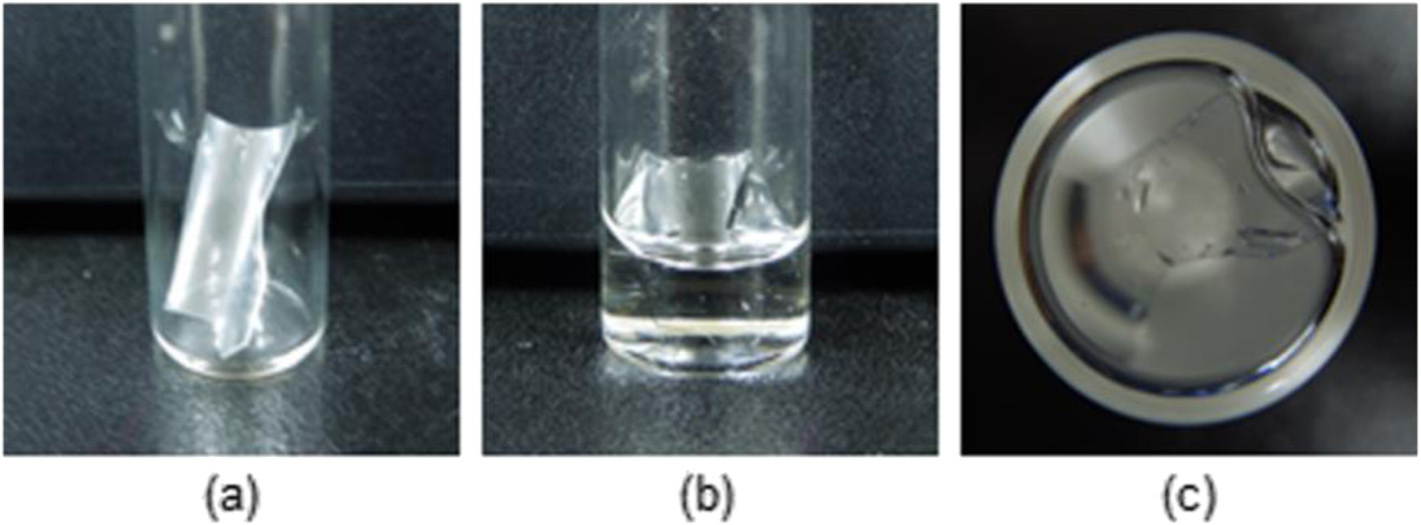
Pictures of polymer films. Pictures of (**a**) poly(AN-co-MAA-co-NIPAM) film; (**b**) side and (**c**) top views in medium

Figure 6 shows the images of swollen poly(AN-co-MAA-co-NIPAM). The prepared films were swollen in media. The mass measurements of swollen films were performed to determine the swelling ratio (Fig. 4b). The swelling ratios of the copolymers were summarized in Table 1. The swelling ratio of poly(AN-co-MAA-co-NIPAM) with a ratio of 25/25/50 was higher than those of poly(AN-co-MAA-co-NIPAM) with ratios of 50/25/50 and 25/50/25, suggesting that the higher hydrophilicities of the copolymer are correlated with increased PNIPAM segment. The swelling ratio increased slightly as the molecular weight increased. Poly(AN-co-MAA) and poly(AN-co-NIPAM) showed lower swelling ratios than poly(AN-co-MAA-co-NIPAM) with a ratio of 25/25/50 because of the higher PAN segment concentration, even though all the copolymers had the same hydrophilic PMAA or PNIPAM segment concentration.

**Fig. 7:**
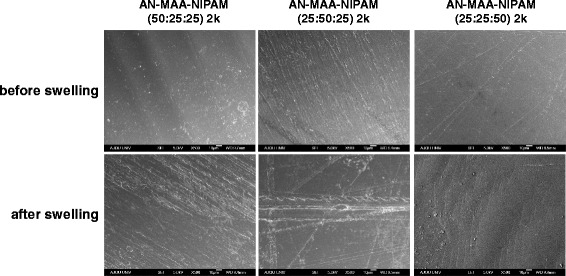
SEM microphotographs. SEM microphotographs before and after swelling of the films prepared from poly(AN-co-MAA-co-NIPAM), poly(AN-co-MAA) and poly(AN-co-NIPAM)

The surface structure of the films was examined by SEM. Figure 7 shows morphologic SEM images of the surface before and after swelling. The surface before swelling seemed to be covered with thin fibers in irregular structure, but non-porous films. After selling the films changed to structures assembled into fibrils of several micrometers in thickness and maintained the non-porous surfaces.

## Conclusion

In this work, we successfully prepared HPAN derivatives with controllable compositions. The properties of the copolymer films depended on the ratio and length of the hydrophobic PAN segment and the hydrophilic PMAA and/or PNIPAM segments. Although future studies will be needed to provide additional biological information associated with cellular studies including cytoxicity, cell growth and proliferation as well as animal experiments, we anticipate that the HPAN derivatives with controllable compositions developed in this study can be potentially used as biomedical hydrogels.
